# Efficacy of pasireotide LAR for acromegaly: a prolonged real-world monocentric study

**DOI:** 10.3389/fendo.2024.1344728

**Published:** 2024-02-01

**Authors:** Vittoria Favero, Benedetta Zampetti, Emanuela I. Carioni, Paolo Dalino Ciaramella, Erika Grossrubatscher, Daniela Dallabonzana, Iacopo Chiodini, Renato Cozzi

**Affiliations:** ^1^ Department of Biotechnology and Translational Medicine, University of Milan, Milan, Italy; ^2^ Unit of Endocrinology, Grande Ospedale Metropolitano Niguarda, Milan, Italy

**Keywords:** acromegaly, pasireotide, somatostatin receptor subtype 5 (SSTR5), insulin-like growth factor 1, growth hormone

## Abstract

**Background:**

Acromegaly is caused by excessive growth hormone (GH) and insulin-like growth factor 1 (IGF1). Medical therapy plays a role as a treatment option for persistent disease after non-curative surgery or as a first-line therapy when surgery is not feasible. Pasireotide-LAR (Pas-LAR) is recommended for patients with acromegaly as second-line treatment.

**Aim:**

To evaluate the patients characteristics predictive of an adequate response to Pas-LAR and the long-term efficacy and safety of the Pas-LAR treatment.

**Methods:**

Data from 19 patients with active acromegaly, who were and resistant or intolerant to first-line medical therapy and were switched to pas-LAR have been retrospectively collected. We compared the baseline clinical and biochemical characteristics of patients who were found to respond to Pas-LAR therapy (responders, n=14) with those of patients who did not respond (non-responders, n=5). We then evaluated the Pas-LAR efficacy and safety during long-term follow-up in responders.

**Results:**

IGF1 normalization occurred in 71.4% of responders after one injection. IGF1 levels, [median(interquartile range) of the upper limit of the normal range (ULN) fold increase] were higher in non-responders compared to responders within the initial month of therapy [1.40(1.30-2.34) vs 0.70(0.55-1.25), respectively, p=0.009] and after three [1.77(1.74-2.29) vs 0.94(0.82-1.13), respectively, p=0.029] and six months [1.68(1.33-1.72) vs 1.00(0.65 -1.28), respectively, p=0.002]. Out of 6 patients with symptomatic headache (all in responder group), 5 and 1 reported the resolution and improvement of headache, respectively, already after the first injection. Median HbA1c levels tended to increase from baseline to 6 months both in responder (36 mMol/Mol to 42 mMol/Mol) and non-responder patients (45 mMol/Mol to 48 mMol/Mol). During long term follow up, in the responder group 2 new patients developed diabetes. Tumor shrinkage was observed in 6 out of 7 evaluated responders, with no cases of size increase during the long-term follow-up.

**Conclusion:**

Pas-LAR is effective and safe and the early identification of responders is possible just after the first administration.

## Introduction

The primary goal of acromegaly treatment is the GH and IGF-1 levels normalization, which leads to disease control, improving life expectancy and reducing comorbidities. Transsphenoidal surgery is the first-line treatment, by which, however, a long-term biochemical control is attained in less than 65% of patients (1–5). Therefore, medical therapy plays a crucial role as an adjunctive treatment option for persistent disease after non-curative surgery and as a first-line therapy when surgery is not feasible (6). Long-acting somatostatin receptor ligands (SRLs), including octreotide long-acting release (LAR) and lanreotide autogel, are considered the first-line medical treatment for acromegaly patients (6).

Pasireotide long-acting release (Pas-LAR) is a somatostatin multireceptor ligand approved as a second-line treatment for acromegaly (7). It can provide effectiveness in patients resistant to first-generation SRLs, potentially due to its higher affinity to somatostatin receptor subtype 5 (SSTR5) (8, 9). Pas-LAR has been suggested to have a safety profile similar to other SRLs, although hyperglycemia and diabetes mellitus occur more frequently (10). However, data regarding the possibility of an early identification of the subjects who will respond to Pas-LAR therapy are largely lacking. Likewise, studies on the effectiveness and safety of Pas-LAR in the real-life are scarcely available in the literature, in particular in patients with a long-term treatment.

The aim of our study, therefore, was to evaluate in active acromegalic patients the clinical characteristics predictive of an adequate response to Pas-LAR and the long-term efficacy and safety of the Pas-LAR treatment.

## Study design, patients, and methods

### Study design

This is a cross-sectional and longitudinal study on retrospective data. We compared the baseline clinical and biochemical characteristics of acromegalic patients who were found to respond to Pas-LAR therapy (responders) with those of acromegalic patients who were found to not respond (non-responders), in order to find out the clinical and/or biochemical characteristics possibly predictive of an adequate response to therapy (cross-sectional arm). We then evaluated the efficacy and safety of Pas-LAR during a long-term follow-up in the responder patients (longitudinal arm).

### Patients and methods

Data from 19 patients (8 women, 11 men, median age 48 years, range 21-69 years; median IGF-1 1.99-fold increase in respect to the upper limit of the normal range – ULN –, range 1.47-2.26) with active acromegaly and resistant to first-generation SRL at high doses and/or intolerant to pegvisomant, who were switched to pas-LAR have been retrospectively collected.

All patients included in the study were referred to the Niguarda Hospital and started treatment with pas-LAR in the period between October 2016 and December 2020. In all patients the shift to pas-LAR was needed due to resistance to first-generation SRL (octreotide LAR or lanreotide autogel) despite at least 6-months with these therapies at maximal doses and/or to intolerance to pegvisomant.

Patients with poorly controlled diabetes mellitus (glycated hemoglobin [HbA1c]>60 mmol/mol) were excluded since they were considered unsuitable to start therapy with pas-LAR.

Fifteen patients were affected with a macroadenoma, while 2 were diagnosed with a microadenoma. In 2 patients, the was no radiological evidence of a pituitary tumor. Eleven participants had persistent disease after neurosurgery, and 2 individuals had also undergone radiosurgery 12 and 24 months prior to commencing Pas-LAR treatment.

**Figure 1:**
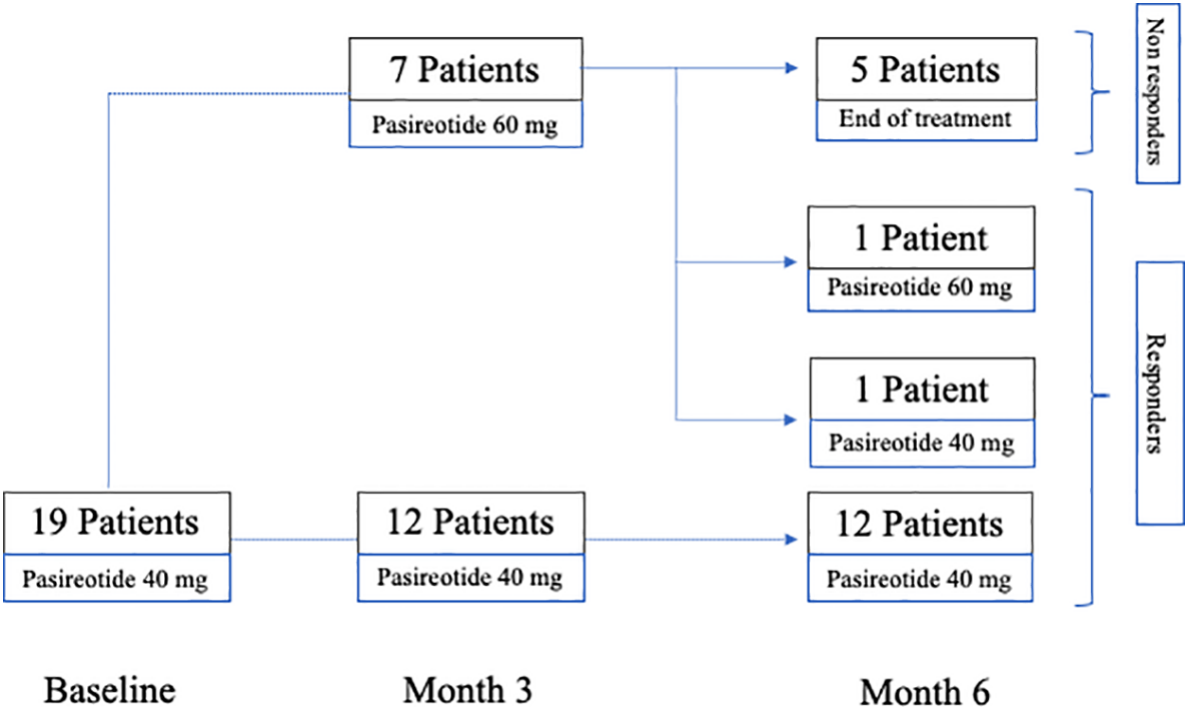
Cross-sectional study profile.

The initial dose of pas-LAR was 40 mg i.m. every 28 days. Pas-LAR was up-titrated to 60 mg if IGF-1 persisted >1.3xULN after 3 months. Biochemical control was defined as age-adjusted IGF-1 <1.3xULN. Treatment was withdrawn if IGF-1 remained ≥1.3xULN after 3 months of treatment with the dose of 60 mg. Patients were considered as “non responders” if at 6 months after the beginning of treatment, despite the Pas-LAR up-titration to 60 mg, IGF-1 value was ≥1.3xULN and as “responders” if IGF-1 value was <1.3xULN (6). The “non responders” group corresponds to the group of patients in whom the medication was withdrawn. These latter patients, therefore, were not included in the longitudinal arm of the study (**Figure 1**).

**Figure 2:**
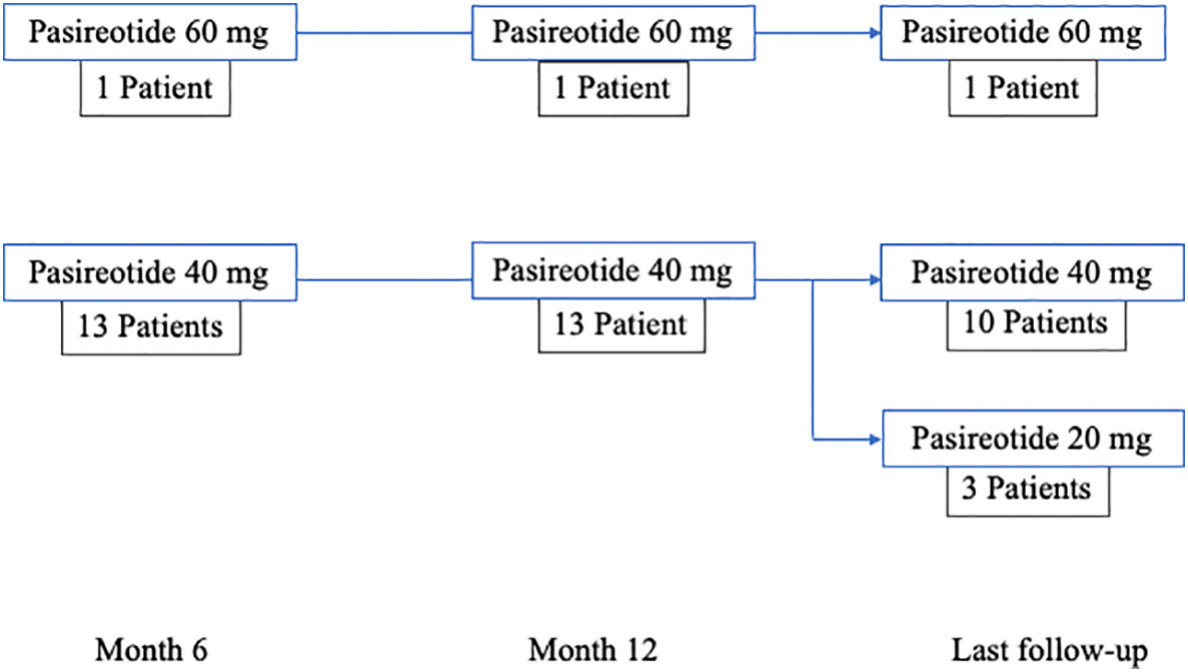
Longitudinal study profile.

The responder patients continued treatment with pas-LAR. The dose was decreased to 20 mg if the IGF-1 levels were <50% ULN. In patients included in the longitudinal arm of the study, data from GH, IGF1 and HbA1c levels were collected after 12 months of treatment and at the last available follow-up (**Figure 2**).

The study was conducted in accordance with the Declaration of Helsinki. Informed consent was obtained from all patients for use of clinical data in research. The investigators adhered to good clinical practice guidelines.

### Statistical analysis

Statistical analysis was performed by JMP (JMP® Pro, Version 16. SAS Institute Inc., Cary, NC, 1989–2021) and GraphPad Prism version 9 (GraphPad Software).

Continuous data are shown as median and interquartile range (IQR). Categorical variables are expressed as numbers and percentages. The comparison of non-parametric continuous data was performed by using Mann-Whitney test or Kruskall-Wallis test followed by Dunn’s *post-hoc* test. The categorical variables were compared by χ2 test or Fisher test, as appropriate. Values of less than 0.05 were considered statistically significant.

## Results

### Cross-sectional arm: characteristics of responder and non-responder patients

The clinical and biochemical characteristics of all patients are reported in **Table 1**. After just one injection, IGF1 levels were <1.3xULN in 10 out of 19 patients (52.6%). After three months of treatment, the dose of Pas-LAR was increased to 60 mg in 7 patients (36.8%). However, even with the higher dose, 5 out of 7 patients did not achieve the disease control, leading to the discontinuation of pas-LAR after 6 months of therapy. Thus, after 6 months of treatment, IGF1 <1.3xULN was achieved in 14 out of 19 patients, and, consequently treatment was withdrawn in 5 out of 19 (26.3%) patients, who were considered as “non-responders” to Pas-LAR therapy (**Figure 1**).

**Table 1:** Patient demographic, clinical and biochemical data before starting pasireotide-LAR.

	All (n=19)	Responders (n=14)	Non responders (n=5)	p value
Demographic				
Women, n (%)	8 (42)	7 (50)	1 (20)	0.33
Age at baseline, years	48 (41-64,5)	47 (42-65)	58 (36-62)	0.71
Radiological characteristics				
Macroadenoma/microadenoma/no visible lesion, n (%)	15/2/2 (79/10.5/10.5)	12/0/2 (85.7/0/14.3)	3/2/0 (60/40/0)	0.11
Treatment prior to pasireotide LAR				
Surgery, n (%)	11 (57.9)	9 (64.3)	3 (60)	0.60
Radiotherapy, n (%)	2 (10.5)	2 (14.3)	0 (0)	1.00
Metabolic status				
Diabetes, n (%)	5 (26.3)	4 (28.6)	1 (20)	0.70
HbA1c, nmol/mol	39 (34.5-45)	36 (34-40)	45 (39-48)	0.09

Categorical variables are reported as N (%), statistical comparison performed by chi-square test or Fisher exact test, as appropriate. Continuous variables are reported as median (IQR) and statistical analysis were performed by Mann-Whitney test.

The comparison of the clinical characteristics at baseline between responders (n=14) and non-responders (n=5) are reported in **Table 1**. Age, radiological characteristics, types of treatments before Pas-LAR, prevalence of diabetes or prediabetes, HbA1c levels, BMI, gender were similar between the two groups.

After just one injection, corresponding to one month of treatment, IGF1 levels were <1.3xULN in 10 out of 14 responders (71.4%), while none of the five non-responders exhibited a reduction to normal values. The IGF1 levels were higher in non-responder patients compared to responder ones even after three and six months (**Table 2**). It is noteworthy that already after only one month of therapy, GH levels were lower in responder subjects than in non-responder ones, even though without reaching the statistical significance. As compared with non-responder patients, GH levels were consistently lower in responder patients at three and six months (**Table 2**).

**Table 2:** Median IGF-1 and GH levels in all patients, in responders and in non responders at baseline and after 1, 3 and 6 months of pasireotide LAR treatment.

	All (n=19)	Responders (n=14)	Non responders (n=5)	p value
IGF-1(x ULN)				
Baseline	1.99 (1.47-2.26)	1.95 (1.51 -2.14)	2.00 (1.35-2.79)	0.889
Month 1	1.14 (0.57-1.42)	0.70 (0.55-1.25)	1.40 (1.30-2.34)	0.009
Month 3	0.94 (0.84-1.30)	0.94 (0.82-1.13)	1.77 (1.74-2.29)	0.029
Month 6	1.27 (0.74-1.46)	1.00 (0.65 -1.28)	1.68 (1.33-1.72)	0.002
GH (ng/mL)				
Baseline	3 (1.8-6.85)	3 (1.92 – 7.42)	3 (1.27-3.9)	0.610
Month 1	1.19 (0.8-1.58)	0.9 (0.8 -1.4)	1.6 (1.29-2.8)	0.152
Month 3	1.4 (0.5-2.6)	1.17 (0.47-1.85)	3.36 (2.63-4.63)	0.048
Month 6	1.37 (0.86-2)	0.85 (0.98 – 1.7)	2 (1.7 – 2.81)	0.034

Continuous variables are reported as median (IQR) and statistical analysis were performed by Mann-Whitney test.

### Longitudinal arm: Pas-LAR efficacy

In responder patients, data were collected even after 12 months of treatment and at the last available follow up (47.1 months, range 12-66). The GH and IGF1 levels had a decreasing trend, though not statistically significant, between month 6 [GH 0.98 (0.85-1.7) ng/mL, IGF-1 1.00 (0.65-1.28)xULN], month 12 [GH 0.85 (0.42-1.82) ng/mL, IGF-1 0.71 (0.57-1.17)xULN] and last follow-up [GH 0.74 (0.29-1.21) ng/mL, IGF-1 0.70 (0.57-0.98)xULN] (p >0.30 for all comparisons. In two patients the dose was increased to 60 mg after three months and continued at the same dose for all the study period in one patient (33 months), while it was reduced to 40 mg after 6 months in the other patient with the disease activity remaining controlled until the last follow up (47 months). In 3 out of 14 patients (21.4%) the dose was reduced to 20 mg after 48, 66 and 72 months of follow up, respectively.

**Figure 3:**
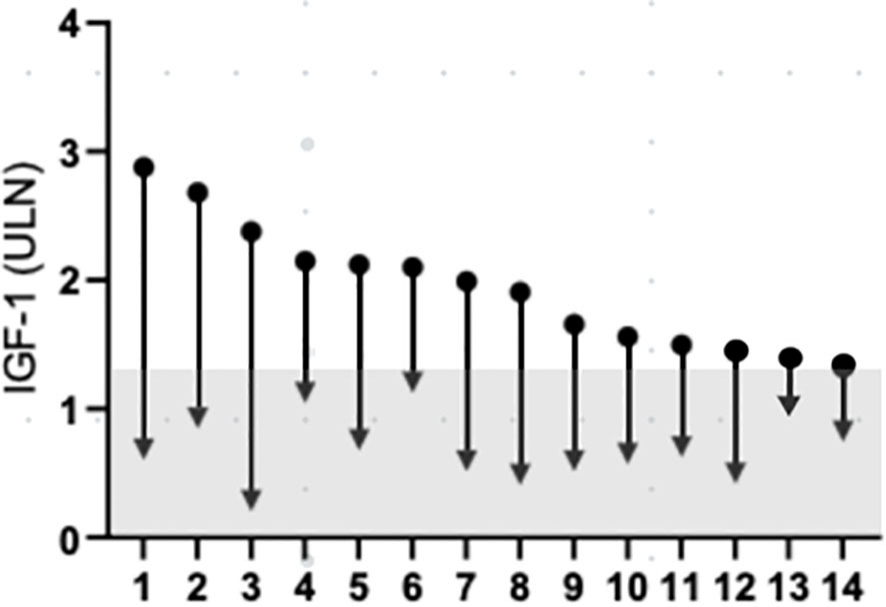
Change IGF-1 concentration, from baseline to last follow-up. Data are individually shown for patients. The grey a represents IGF-1 values below 1.3xULN. IGF-1, insulin-like growth factor 1; ULN, upper limit of normal.

The individual data on the reduction of IGF-1 and GH levels in responder patients are depicted in **Figure 3**. The last acromegaly consensus considered biochemical remission as normalized IGF-I values (11). On the basis of this criterion at 1, 3, 6, 12 months and at last follow-up the prevalence of patients without normalized IGF-1 levels (i.e. IGF-1 levels ≤1xULN) among responder patients was 36.7% (pt #6, 8, 10, 12, 14 in **Figure 3**), 35.7% (pt #6, 8, 10, 12, 14 in **Figure 3**), 42.8% (pt #1, 6, 8, 10, 13, 14 in **Figure 3**), 28.6% (pt #1, 6, 8, 14 in **Figure 3**) and 21.4% (pt #1, 6, 14 in **Figure 3**), respectively.

In one patient, who had undergone irradiation, therapy with pas-LAR was reduced to 20 mg and temporarily stopped due to the normalization of IGF1 levels (reduced by more than 50% of the ULN in Pas-LAR 20 mg). However, after pas-LAR was withdrawn, the patient experienced a recurrence of headaches and the presence of abnormal IGF1 levels. Pas-LAR was then reintroduced, leading to the resolution of headaches and the IGF1 levels control.

Overall, 6 patients complained of acromegalic headache (symptomatic score: 3/3 in 5 and 2/3 in the last), which was resistant to medical treatment before starting pas-LAR. Five patients reported the resolution of the symptoms already after the first injection. The remaining patient reported an improvement of headache. All these patients were in the responder group. The resolution of acromegalic headache persisted consistently throughout the long-term follow-up period.

Tumor shrinkage, defined as a reduction of more than 20% of the basal volume (20-35%), was observed in 6 out of 7 patients who had magnetic resonance imaging data available at the last follow-up, between 12 and 36 months after starting Pas-LAR. Throughout the long-term follow-up, there were no cases of tumor size increase.

### Longitudinal arm: Pas-LAR safety

**Figure 4:**
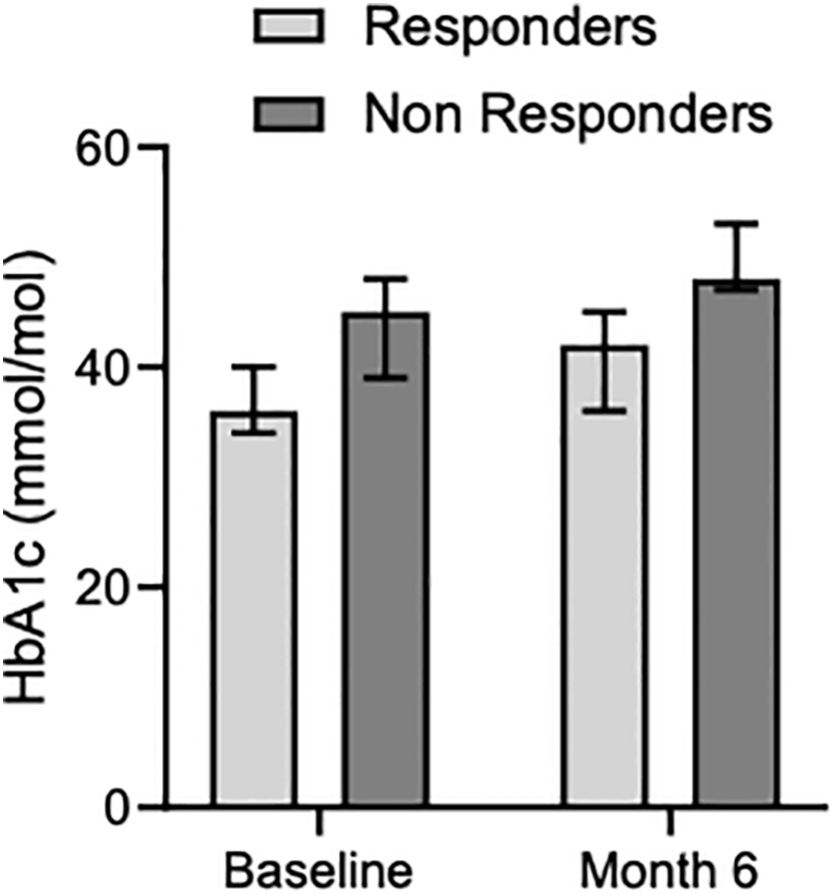
Median glycated hemoglobin levels in responders and non-responders at baseline and month 6.

Median HbA1c levels increased both in responder and non-responder patients from baseline [36 mMol/Mol (34-40) and 45 mMol/Mol (39-48), respectively] to 6 months [42 mMol/Mol (36-45) and 48 mMol/Mol (47-53), respectively] (**Table 3**, **Figure 4**). Before enrollment, 5 out of 19 patients had diabetes. Among them, 4 patients were on medical treatment with metformin, and one patient had well-controlled diabetes without medications. Three out of the 4 patients with drug-treated diabetes were in the responder group and one in the non-responder group. The patient with controlled diabetes only with diet had to start metformin soon after Pas-LAR was introduced and few months later a GLP1 receptor agonist was started. At the six-month evaluation, 4 new patients (3 in the non-responder group) developed diabetes. Among patients in the responder group, 4 patients had diabetes at baseline, and 1 patient developed diabetes within the first 6 months of treatment. Between 6 and 12 months of therapy no new cases of diabetes were observed. At the last follow-up, additional 2 patients had developed diabetes. All the patients with diabetes at baseline required an antidiabetic therapy during the follow-up period. Among the 4 patients initially treated with only metformin, one patient had a DPP4 inhibitor added, one patient had a GLP1 receptor agonist added, and two patients had insulin and a GLP1 receptor agonist added.

**Table 3:** Glycated hemoglobin values at baseline and during long term follow up.

	Responders (n=14)			
HbA1c (mmol/mol)	Mean (IQR)	< 42 mmol/mol (n, %)	42 – 48 mmol/mol (n, %)	>48 mmol/mol (n, %)
Baseline	39 (34.5 – 45)	12 (85.8)	1 (7.1)	1 (7.1)
Month 6	44.5 (39.25-49.25)	8 (57.2)	4 (28.6)	2 (14.2)
Month 12	41 (38-45.75)	7 (50)	5 (35.8)	2 (14.2)
Last follow up	41.5 (36.5-47)	7 (50)	4 (28.6)	3 (21.4)

Categorical variables are reported as n (%) and continuous variables are reported as median (IQR).

Hba1c, glycated hemoglobin; IQR, interquartile range.

Pas-LAR medication was discontinued in 2 out of 14 patients. One patient discontinued the medication after 36 months due to uncontrolled diabetes and poor adherence to diabetes medication, while the other patient discontinued after 60 months due to QTc interval elongation at electrocardiograph exam during concomitant amiodarone treatment. In both cases, the last follow-up time was considered just before they stopped taking the medication. In the responder group, no patients had to stop the medication during the long-term follow-up period due to uncontrolled disease.

## Discussion

The present data show the potential for the early identification of responsive patients to PasLAR therapy and document the long-term effectiveness and safety of this drug for treating acromegaly.

This study comprehensively assesses the response trends in terms of GH and IGF-1 levels to Pas-LAR by considering both patients who continued and those who discontinued therapy due to its ineffectiveness. According to the present data, in patients with active acromegaly and resistant to first-generation SRL at high doses and/or intolerant to pegvisomant, Pas-LAR has proven to be effective in 52.6% of cases. Importantly, the 71.4% of responder patients experience the IGF1 normalization in one month after the initial injection, as mirrored by the observation that, after one month of treatment, responder patients exhibit lower IGF-1 values compared to non-responders. However, there is a slight delay in the response if GH values are considered, which are lower in responder patients only at the third month of therapy compared to non-responder ones. Within just three months of therapy, most responder patients can be identified, and the dosage escalation to 60 mg is needed in a minority of subjects. Remarkably, indeed, 12 out of 14 responder patients (85.7%) achieved the disease control after three months of therapy and maintained it for the entire duration of the study without requiring a dosage increase during the follow-up period. On the other hand, all non-responder patients, needed to increase the dosage to 60 mg/day after 3 months, but even so, they did not achieve control of the disease. It is interesting to note that in two responder patients, it was necessary to increase the dosage after 3 months, and in one case, disease normalization was achieved at 6 months, allowing the dose to be reduced. In the second patient it was necessary to maintain a 60 mg/day dose throughout the entire observation period.

The data from the longitudinal arm of the study shows that the disease control remained stable over time. Notably, in some cases, reducing the dose by half proved effective in maintaining IGF1 normalization. This finding suggests that no escape from therapy has to be feared, and that patients who initially respond to the Pas-LAR treatment will continue to respond over time. According to our results from the longitudinal study, GH and IGF-I levels continue to decrease over time with Pas-LAR treatment, indicating that the clinical benefit of this drug is maintained and could be further improved with long-term use. This was also observed in a recent study by Galdelha and coauthors, in which a larger proportion of patients achieved the biochemical control (as defined as IGF-I ≤ULN) at the last follow-up visit compared to the disease activity after the first 12 months of therapy (12). A similar observation was made in a retrospective study on 19 acromegaly patients treated with Pas-LAR for an average of 50 months. In this study 11 patients achieved IGF-I normalization within 12 months, and additional 6 patients achieved GH secretion normalization after 12 months (13).

The present data also show that the Pas-LAR dosage might be reduced over time while maintaining disease control. This possibility of reducing Pas-LAR treatment was initially suggested in a small study by Giampietro and coauthors, which reported three cases of acromegalic patients in whom pas-LAR treatment was reduced to 20 mg during a long-term follow-up (14). More recently, Marques and coauthors showed that 40% of patients were able to reduce Pas-LAR dose, with the IGF-I levels showing a mild increase but remaining within the normal range after a median of 39 months in the early responders (1-3 months of treatment, n=11) and 17 months in the late responders (after ≥4 months, n=9) (15). This phenomenon of IGF-I levels over-suppression has been previously described by Shimon (16) and it could be due to the fact that during Pas-LAR therapy the somatostatin receptors are not desensitized but, on the contrary, they may become more ligand-responsive over the years (13).

Consistent with previous studies on Pas-LAR, in our study the occurrence of hyperglycemia was common, but it was mostly mild and manageable. Glucose metabolism is often worsened in patients treated with Pas-LAR and it may be associated with the dosage used. Nevertheless, the control of acromegaly impacts the development of hyperglycemia, as GH and IGF-I play a major role in glucose metabolism regulation (3, 17). In our series, only one patient discontinued treatment due to hyperglycemia. During long-term follow-up, we observed the increase of HbA1c despite an increased number of medications used for the management of hyperglycemia and despite a better acromegalic disease control. This suggests that in acromegalic patients treated with pas-LAR, the occurrence of hyperglycemia is related to the treatment and not to the disease activity.

It is known that the first-generation somatostatin analogues have an important beneficial effect on headache. However, they require multiple daily injections, with the headache reappearing after few hours, while with Pas-LAR the effect is prolonged with the complete disappearance of this symptom. Furthermore, it is has been reported that patients with severe headaches, who are not responsive to first-generation SRL therapy, are likely to experience an improvement in headache symptoms after starting Pas-LAR treatment (18). We observed a dramatic effect of Pas-LAR on headache, with the resolution of symptoms occurring soon after the first injection in 5 out of 6 patients and the headache improvement in the remaining patient. This striking effect on the headache control was also reported in another study on 18 patients, in which the headache was resolved and improved in 11 and 3 patients, respectively (19). The mechanisms underlying the beneficial effect of the somatostatin analogues on headache are still largely unknown. We know that the somatostatin receptor subtype 2 are located in brain areas potentially crucial for cluster headache due to their interconnections with the opiodergic system. Other data seem to point toward a possible effect of cytokines or vasoactive substances in the pathogenesis of headache in some cases. Finally, this important effect on headache may be related to the tumor expression of a truncated somatostatin receptor 5 variant, as described in two cases of patients with severe headaches and no biochemical effects of octreotide, but a good response to Pas-LAR (20). Therefore, in agreement with other authors (18), we suggest that Pas-LAR could be considered as a second-line therapy for patients with headache not responsive to first-generation SRL therapy.

Finally, in our patient cohort we did not observe any cases of increased adenoma size. Instead, we found an effect on reducing tumor mass during long-term therapy in a significative number of patients. This finding deserves to be confirmed in a larger number of patients since some patients included in the present study, had already obtained a tumor shrinkage due to the previous first-generation SRL treatment.

Beside the sample size, our study has other limitations. Firstly, the retrospective design implies that some unknown confounding factors could have played a role. Secondly, PAS-LAR treatment in non-responder patients was withdrawn only after 6 months of ineffective therapy, which may have prevented us to observe a delayed response in some of the patients in whom treatment was discontinued. Finally, we were not able to provide data on the T2 intensity at magnetic resonance or granulation pattern at histopathology as predictors of response to Pas-LAR, which could have been more informative (21).

Notwithstanding these limitations, the present study suggests that in acromegalic patients resistant to first-generation SRL at high doses and/or intolerant to pegvisomant, Pas-LAR is an effective and safe drug. Moreover, the Pas-LAR peculiar mechanism of action allows for the early identification of responsive patients just after the first administration and the effectiveness of this drug against headache has to be considered an important advantage.

## Data availability statement

The raw data supporting the conclusions of this article will be made available by the authors, without undue reservation.

## Ethics statement

Ethical approval was not required for the studies involving humans because the retrospective nature of the study. The studies were conducted in accordance with the local legislation and institutional requirements. Written informed consent for participation was not required from the participants or the participants’ legal guardians/next of kin in accordance with the national legislation and institutional requirements because this study was conducted in adherence to good clinical practice guidelines.

## Author contributions

VF: Data curation, Formal analysis, Writing – original draft, Writing – review & editing. BZ: Data curation, Writing – review & editing. EC: Data curation, Writing – review & editing. PD: Data curation, Writing – review & editing. EG: Data curation, Writing – review & editing. DD: Data curation, Writing – review & editing. IC: Formal analysis, Methodology, Supervision, Validation, Writing – original draft. RC: Conceptualization, Formal analysis, Investigation, Project administration, Supervision, Validation, Visualization, Writing – original draft.
